# Strong Neutralizing Antibody Responses to SARS-CoV-2 Variants Following a Single Vaccine Dose in Subjects With Previous SARS-CoV-2 Infection

**DOI:** 10.1093/ofid/ofac625

**Published:** 2022-11-19

**Authors:** Nina Ekström, Anu Haveri, Anna Solastie, Camilla Virta, Pamela Österlund, Hanna Nohynek, Tuomo Nieminen, Lauri Ivaska, Paula A Tähtinen, Johanna Lempainen, Pinja Jalkanen, Ilkka Julkunen, Arto A Palmu, Merit Melin

**Affiliations:** Expert Microbiology Unit, Department of Health Security, Finnish Institute for Health and Welfare, Helsinki, Finland; Expert Microbiology Unit, Department of Health Security, Finnish Institute for Health and Welfare, Helsinki, Finland; Expert Microbiology Unit, Department of Health Security, Finnish Institute for Health and Welfare, Helsinki, Finland; Expert Microbiology Unit, Department of Health Security, Finnish Institute for Health and Welfare, Helsinki, Finland; Expert Microbiology Unit, Department of Health Security, Finnish Institute for Health and Welfare, Helsinki, Finland; Infectious Disease Control and Vaccinations Unit, Department of Health Security, Finnish Institute for Health and Welfare, Helsinki, Finland; Data and Analytics Unit, Department of Knowledge Brokers, Finnish Institute for Health and Welfare, Helsinki, Finland; Department of Pediatrics and Adolescent Medicine, Turku University Hospital, Turku, Finland; Department of Pediatrics and Adolescent Medicine, Turku University Hospital, Turku, Finland; Department of Pediatrics and Adolescent Medicine, Turku University Hospital, Turku, Finland; Clinical Microbiology, Turku University Hospital, Turku, Finland; Immunogenetics Laboratory, Institute of Biomedicine, University of Turku, Turku, Finland; Infection and Immunity, Institute of Biomedicine, University of Turku, Turku, Finland; Clinical Microbiology, Turku University Hospital, Turku, Finland; Infection and Immunity, Institute of Biomedicine, University of Turku, Turku, Finland; Interventions Unit, Department of Public Health and Welfare, Finnish Institute for Health and Welfare, Tampere, Finland; Expert Microbiology Unit, Department of Health Security, Finnish Institute for Health and Welfare, Helsinki, Finland

**Keywords:** hybrid immunity, neutralizing antibodies, previous infection, SARS-CoV-2, variants of concern

## Abstract

**Background:**

Previous severe acute respiratory syndrome coronavirus 2 (SARS-CoV-2) infection primes the immune system; thus individuals who have recovered from infection have enhanced immune responses to subsequent vaccination (hybrid immunity). However, it remains unclear how well hybrid immunity induced by severe or mild infection can cross-neutralize emerging variants. We aimed to compare the strength and breadth of antibody responses in vaccinated recovered and uninfected subjects.

**Methods:**

We measured spike-specific immunoglobulin (Ig)G and neutralizing antibodies (NAbs) from vaccinated subjects including 320 with hybrid immunity and 20 without previous infection. From 29 subjects with a previous severe or mild infection, we also measured NAb responses against Alpha (B.1.1.7), Beta (B.1.351), Delta (B.1.617.2), and Omicron (B.1.1.529/BA.1) variants following vaccination.

**Results:**

A single vaccine dose induced 2-fold higher anti-spike IgG concentrations and up to 4-fold higher neutralizing potency of antibodies in subjects with a previous infection compared with vaccinated subjects without a previous infection. Hybrid immunity was more enhanced after a severe than a mild infection, with sequentially decreasing NAb titers against Alpha, Beta, Delta, and Omicron variants. We found similar IgG concentrations in subjects with a previous infection after 1 or 2 vaccine doses.

**Conclusions:**

Hybrid immunity induced strong IgG responses, particularly after severe infection. However, the NAb titers were low against heterologous variants, especially against Omicron.

Infection with severe acute respiratory syndrome coronavirus 2 (SARS-CoV-2) induces antibodies to the viral spike glycoprotein (S), which is also the target of coronavirus disease 2019 (COVID-19) vaccines. The generation of neutralizing antibodies (NAbs) that specifically target the receptor-binding domain (RBD) of the S protein is considered essential in controlling SARS-CoV-2 infection. We and others have previously shown that circulating antibodies gradually decrease following wild-type (WT) infection but that NAbs are sustained at a detectable level for up to 15 months [1, 2]. However, antibody-mediated immunity induced by infection with the ancestral virus is reduced against SARS-CoV-2 variants with immune escape mutations, as only part of the NAbs can bind to the RBD of these variants [3, 4]. The Omicron variant (B.1.1.529) especially has acquired new mutations in the RBD [5, 6], resulting in evolutionary Omicron sublineages (BA.1, BA.2, BA.3, BA.4, and BA.5), which have given rise to major epidemic waves worldwide, causing breakthrough infections also in vaccinated individuals.

COVID-19 vaccination after recovery from SARS-CoV-2 infection (hybrid immunity) has been reported to induce comparable or higher S-specific antibody levels and NAb titers than in twice-vaccinated SARS-CoV-2-naïve individuals [7–12]. In addition, vaccination has been shown to elicit immunity with broader specificity and increase the neutralization potency against SARS-CoV-2 variants in previously infected individuals [13, 14]. Previous studies have shown no increase in circulating antibodies, neutralizing titers, or antigen-specific memory B cells after >1 dose of vaccine in those with previous infection [8, 15, 16]. Hybrid immunity-induced antibody concentrations and NAbs have been shown to decline with time but remain at a higher level than in uninfected vaccinated individuals for at least 3 months [8, 11, 17]. Furthermore, hybrid immunity has been associated with a somewhat lower risk of reinfection and hospitalization compared with immunity induced solely by previous infection [18–20]. To better understand the level of protection hybrid immunity provides against different SARS-CoV-2 variants, including Omicron (B.1.1.529), we compared the strength and breadth of IgG and NAb responses induced by hybrid immunity to vaccination or infection and assessed how the difference in disease severity affects the development of hybrid immunity.

## METHODS

### Study Design

**Figure 1.**
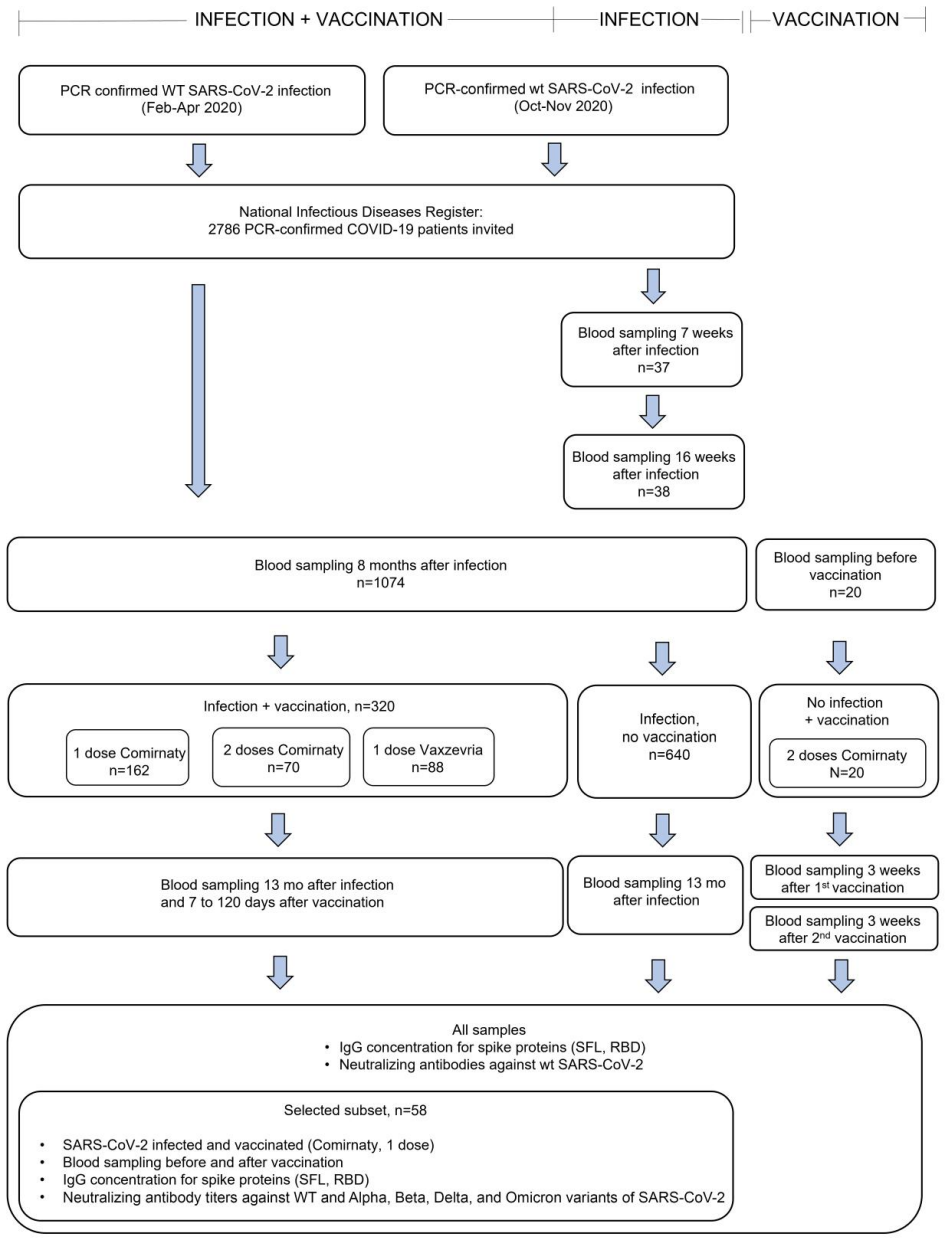
Selection of study subjects with or without a previous SARS-CoV-2 infection and with or without a subsequent SARS-CoV-2 vaccination and selection of serum samples for the determination of spike protein–specific IgG antibody concentration and neutralizing antibodies. Abbreviations: COVID-19, coronavirus disease 2019; IgG, immunoglobulin G; PCR, polymerase chain reaction; SARS-CoV-2, severe acute respiratory syndrome coronavirus 2; WT, wild-type.

This was an observational study assessing immune responses induced by SARS-CoV-2 infection and vaccination. Vaccinations were administered according to the Finnish national COVID-19 vaccination program starting in December 2020. We collected blood samples after infection and vaccination (Figure 1), separated the specimens by centrifugation, and stored aliquoted serum at −20°C or −70°C. We defined the COVID-19 severity as severe or mild. Severe infection was defined as laboratory-confirmed COVID-19 requiring hospital treatment based on data collected from the hospital discharge register (Care Register for Health Care), and mild infection as laboratory-confirmed COVID-19 without documentation of hospital treatment. We collected the demographics, clinical characteristics, and COVID-19 vaccination history of the participants from the National Infectious Disease Register and the National Vaccination Register (Supplementary Table 1).

### Samples From Infected, Vaccinated Subjects

We identified 2586 subjects ≥18 years of age with polymerase chain reaction (PCR)–confirmed SARS-CoV-2 infection between February and April 2020 in the National Infectious Disease Register and invited them to participate [1]. The participants were infected when the B, B.1, and B.1.1 lineage viruses (hereafter referred to as wild-type [WT]) were introduced into Finland [22]. We collected a blood sample for determination of SARS-CoV-2-specific serum antibodies from 1074 subjects at 8 months (median [range], 7.7 [6.1–11.4] months) and 13 months (median [range], 12.9 [11.8–17.3] months) after SARS-CoV-2 infection. In this study, we included participants who had received 1 dose of COVID-19 vaccine (BNT162b2, Comirnaty, Pfizer-BioNTech, n = 162, or ChAdOx1, Vaxzevria, AstraZeneca, n = 88) 7–90 days or 2 doses of Comirnaty (n = 70) 7–120 days before the 13-month sampling (Figure 1; Supplementary Table 1).

We selected paired serum samples from 30/55 of the study participants with a previous SARS-CoV-2 infection followed by 1 dose of Comirnaty 22–90 days before sampling for the determination of NAb titers against WT virus and 4 variants: Alpha (B.1.1.7), Beta (B.1.351), Delta (B.1.617.2), and Omicron (B.1.1.529/BA.1) (Figure 1). We first selected 15/15 subjects with a sample taken 59–90 days after vaccination; 8/15 of these subjects had previous severe disease. We then selected 15/40 subjects with a sample taken 21–30 days after vaccination by matching disease severity, age, and gender to the first group. One subject with mild disease was afterwards excluded due to the administration of 2 doses of Comirnaty before sampling.

### Samples From Infected, Not Vaccinated Subjects

We collected serum samples from 640 subjects ≥18 years of age 13 months after PCR-confirmed WT SARS-CoV-2 infection between February and April 2020 with no documentation of COVID-19 vaccination before sampling (Figure 1; Supplementary Table 1) [1]. We excluded subjects with a >30% increase in IgG antibodies to nucleoprotein or spike protein between samples taken at 8 and 13 months after infection. We additionally collected serum samples from 38 subjects with no COVID-19 vaccination history and with a previous WT SARS-CoV-2 infection (between October and November 2020) at a median (range) of 51 (19–59) days and 118 (90–148) days after infection (Figure 1; Supplementary Table 1).

### Samples From Vaccinated, Uninfected Subjects

Serum samples from subjects with no history of previous SARS-CoV-2 infection and who had received 2 doses of COVID-19 vaccine (Comirnaty) were obtained from Turku University Hospital (TUH, Turku, Finland). Blood samples from 20 subjects were collected at a median (range) of 21 (18–21) days after the first vaccine dose and 27 (18–29) days after the second vaccine dose (at 39–50 days after the first vaccine dose).

### Patient Consent

The study protocol of the serological population study of the coronavirus epidemic was approved by the ethical committee of the Hospital District of Helsinki and Uusimaa (HUS/1137/2020). The study protocol of the study of COVID-19 infections in hospital personnel [21] was approved by the ethical committee of the Southwest Finland health district (ETMK 19/1801/2020) and by the Finnish Medicines Agency Fimea as the regulatory authority (European Union Drug Regulation Authority's clinical trials database code 2021-004419-14). Written informed consent was obtained from all participants before sampling.

## METHODS

### SARS-CoV-2 Fluorescent Multiplex Immunoassay

We measured the concentration of serum IgG antibodies to WT spike glycoprotein of SARS-CoV-2 (S-IgG; full-length spike protein [SFL-IgG] and receptor binding domain of spike protein [RBD-IgG]) with a fluorescent multiplex immunoassay (FMIA) [23]. Antibody concentrations are given as WHO international binding antibody units (BAU)/mL [24]. IgG SARS-CoV2 FMIA is an accredited assay at the Finnish Institute of Health and Welfare, which is a testing laboratory T077 accredited by the FINAS Finnish Accreditation Service, accreditation requirement SFS-EN ISO/IEC 17025.

### SARS-CoV-2 Microneutralization Test

We performed a live-virus microneutralization test (MNT) as previously described [1, 3, 25]. All samples were screened with WT virus (B lineage) (Supplementary Table 2) for NAb positivity. NAb titers for selected samples were analyzed with 4 SARS-CoV-2 variants isolated in Finland during 2021 (Alpha [B.1.1.7], Beta [B.1.351], Delta [B.1.617.2], and Omicron [B.1.1.529/BA.1]) (Supplementary Table 2). WT virus isolation and propagation were performed in African green monkey kidney epithelial (VeroE6) cells [25]. All variant viruses were isolated and propagated in VeroE6-TMPRSS2-H10 cells [26] and further propagated in VeroE6 cells for MNT. A tissue culture infectious dose 50% (TCID_50_)/mL titer was determined with an end-point dilution assay with the same incubation times as MNT for all viruses to achieve a comparable virus concentration (100 TCID_50_ per well) among the different virus strains.

Results were expressed as MNT titers corresponding to the reciprocal of the serum dilution that inhibited 50% of SARS-CoV-2 infection. A titer ≥6 was considered positive, 4 borderline, and <4 negative. Borderline values were confirmed with biological repeats. A titer of 192, 96, 8, 32, and <4 was measured for the WHO International Standard (NIBSC 20/136) [24] for the WT virus, Alpha, Beta, Delta, and Omicron BA.1 variants, respectively.

The neutralizing potency (neutralizing activity adjusted for IgG antibody concentration) of serum was calculated as the ratio of neutralizing activity (MNT titer) to anti-spike IgG antibody concentration (BAU/mL) and was determined for samples taken following hybrid immunity (selected subgroup, n = 29) and following 2 doses of vaccine (n = 20).

### Statistical Methods

We calculated the geometric mean concentrations (GMCs) and titers (GMTs) with 95% CIs for IgG and NAbs, respectively. For GMT calculation, MNT titers <4 were assigned a titer value of 2. We assessed the statistical differences in antibody levels, neutralizing antibody titers, and neutralizing potencies between the groups with the Wilcoxon rank-sum test. We set the statistical significance level of difference to *P* < .05. We performed the statistical analyses using GraphPad (version 9) and R (version 4.0.4) with Rstudio (version 1.4.1106).

## RESULTS

### Spike-Specific and Neutralizing Antibodies Following Hybrid Immunity or Vaccination

We measured spike-specific antibodies in subjects previously infected with SARS-CoV-2 following 1 dose of COVID-19 vaccine. We found that before vaccination at 8 months after infection, 97% of the subjects were positive for S-IgG and 89% had NAbs against WT SARS-CoV-2. A single vaccine dose at a median (range) of 351 (250–424) days after infection induced >20-fold higher IgG concentrations in previously infected compared with uninfected subjects (Table 1; Supplementary Figure 2).

**Table 1.** Geometric Mean Anti-SARS-CoV-2 Spike Protein IgG Concentrations GMC (95% CI) Expressed as Binding Antibody Units/mL for Wild-Type Spike Proteins, Receptor Binding Domain of Spike, and Full-Length Spike Protein, Percentage of Positive Samples for Spike Protein IgG and Neutralizing Antibodies in Vaccinated and Unvaccinated Subjects With a Previous SARS-CoV-2 Infection and in Uninfected Vaccinated Subjects Measured From Samples Taken After SARS-CoV-2 Infection, Stratified by Days of Serum Sample Collection Since Last Vaccination; Nabs Were Measured Against WT SARS-CoV-2 Virus

Infection and Vaccination Status, Days From Vaccination or Infection		GMC (95% CI)				% Positive	
	No.	RDB-IgG		SFL-IgG		S-IgG	NAb
Infected, Comirnaty 1 dose	**…**	**…**	**…**	**…**	**…**	**…**	**…**
7–30	102	120	(94–154)	238	(189–300)	100	98
31–60	50	101	(75–137)	187	(137–256)	100	98
61–90	10	61	(20–186)	155	(71–336)	100	100
Infected, Vaxzevria 1 dose	…	…	…	…	…	…	…
7–30	29	78	(53–115)	138	(95–200)	100	100
31–60	37	44	(27–74)	68	(40–117)	100	97
61–90	22	40	(24–65)	51	(30–89)	100	95
Infected, Comirnaty, 2 doses	…	…	…	…	…	…	…
7–30**^a^**	12	146	(93–229)	253	(168–382)	100	100
61–90**^b^**	29	82	(59–115)	137	(100–187)	100	100
91–120**^b^**	30	76	(60–96)	112	(87–146)	100	100
Infected, not vaccinated	…	…	…	…	…	…	…
14–60	37	2.5	(1.0–5.9)	2.8	(1.3–6.0)	89	92
90–148	38	1.7	(0.79–3.5)	2.1	(1.1–4.2)	89	89
362–448	640	1.7	(1.6–1.9)	2.3	(2.1–2.5)	97	90
Not infected, Comirnaty	…	…	…	…	…	…	…
14–30 (after 1 dose)	20	3.8	(2.1–7.1)	9.2	(5.7–15)	100	100
14–30 (after 2 doses)	20	63	(42–96)	95	(64–142)	100	100

Abbreviations: IgG, immunoglobulin G; NAb, neutralizing antibody; RBD, receptor-binding domain; SARS-CoV-2, severe acute respiratory syndrome coronavirus 2; SFL, full-length spike protein; S-IgG, spike protein IgG; WT, wild-type.

a First and second vaccines at a median (range) of 84 (49–88) days apart.

b First and second vaccines at a median (range) of 21 (19–28) days apart.

**Figure 2.**
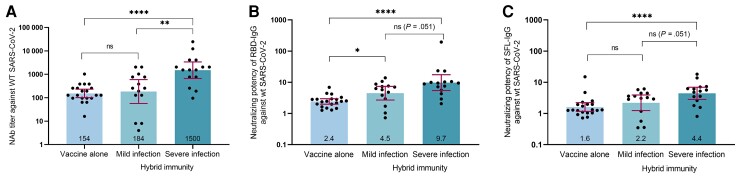
Mean NAb titers against WT SARS-CoV-2 (*A*), mean neutralizing potency against WT SARS-CoV-2 of IgG antibodies targeting RBD (*B*), and SFL (*C*) in the selected subgroup of subjects only vaccinated (n = 20) or with hybrid immunity after a severe (n = 15) or mild infection (n = 14.) The height of the bars represents the geometric mean, and whiskers represent 95% confidence intervals within the group. Geometric mean values are shown in the bottom of the bars. Statistically significant differences between groups are indicated with asterisks (Wilcoxon rank-sum test): *P < .05; **P < .01; ***P < .001. Abbreviations: IgG, immunoglobulin G; NAb, neutralizing antibody; RBD, receptor-binding domain; SARS-CoV-2, severe acute respiratory syndrome coronavirus 2; SFL, full-length spike protein; WT, wild-type.

We compared the antibody concentrations in subjects with previous infection after 1 vaccine dose (Comirnaty or Vaxzevria) with those of subjects without infection after 2 doses of Comirnaty 7–30 days after the last dose. We found 2-fold higher S-IgG concentrations after infection followed by 1 dose of Comirnaty compared with 2 doses of Comirnaty alone (*P* < .001) (Table 1; Supplementary Figure 2). In subjects receiving 1 dose of Vaxzevria following infection, we found similar or slightly higher S-IgG concentrations compared with subjects who received 2 doses of vaccine (Comirnaty; *P* = .237 and 0.04, for RBD- and SFL-IgG, respectively) (Table 1; Supplementary Figure 2). We found a trend for higher mean IgG concentrations in subjects with hybrid immunity following 1 dose of Comirnaty than Vaxzevria (Table 1; Supplementary Figure 1). A high percentage of subjects with hybrid immunity (98%) and vaccinated subjects without previous infection (100%) had NAbs against WT SARS-CoV-2 (Table 1). However, when we compared the NAb titers and neutralizing potency of the antibodies against WT SARS-CoV-2 in the age- and gender-matched subgroup, we found that >8-fold and 1.4–4-fold higher mean NAb titers and neutralizing potency of antibodies, respectively, were reached following hybrid immunity compared with 2 doses of vaccine alone (Figure 2). Further, the greater difference in NAb titers and neutralizing potencies between vaccinated subjects and subjects with hybrid immunity after severe disease compared to subjects with mild disease suggests that hybrid immunity is more enhanced following severe than mild infection (Figure 2). At 90 days following hybrid immunity, the mean antibody levels had decreased, but 97% of the subjects still had NAbs (Table 1).

We assessed the effect of a second vaccine dose on hybrid immunity by measuring IgG concentrations and NAbs after 2 doses of Comirnaty. The second dose was administered either with a short (median [range], 21 [19–28] days; n = 59) or long (median [range], 84 [49–88] days; n = 12) dosing interval at a median of 277 days after infection. We found comparable S-IgG concentrations after the first and second doses and with either a short or long dosing interval, suggesting that in hybrid immunity the second vaccine dose did not further enhance S-IgG levels (*P* > .05) (Table 1; Supplementary Figure 1). To take into account the larger proportion of subjects with severe disease in the 1-dose compared with the 2-dose group, we included only subjects with mild disease in the comparison. In line with high antibody levels induced by hybrid immunity, the proportion of subjects with NAbs was high after 1 (98%) and 2 doses of the vaccine (100%) (Table 1).

### Neutralization of SARS-CoV-2 Variants After Hybrid Immunity

**Figure 3.**
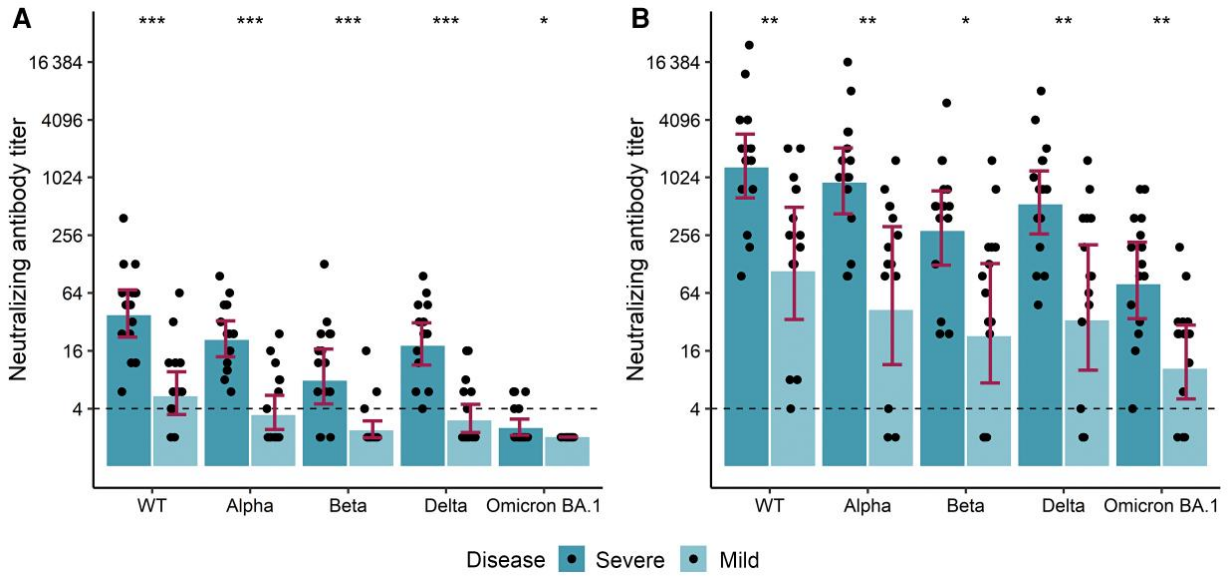
Neutralizing antibody titers to wild-type virus and the Alpha (*B*.1.1.7), Beta (*B*.1.351), Delta (*B*.1.617.2), and Omicron (*B*.1.1.529/BA.1) variants in the severe (n = 15) and mild (n = 14) infection groups 8 months after infection and before vaccination (*A*) and 1–3 months after 1 dose of COVID-19 vaccine (Comirnaty) administered 1 year after infection (*B*). The height of the bars represents the median, and whiskers represent the interquartile range within infection group. The dashed line marks the threshold for a positive result (titer 4). Statistically significant differences between groups with mild and severe infection are indicated with asterisks (Wilcoxon rank-sum test): *P < .05, **P < .01, ***P < .001. Abbreviations: COVID-19, coronavirus disease 2019; WT, wild-type.

A subset of participants with a previous severe (n = 15) or mild (n = 14) WT SARS-CoV-2 infection were selected for NAb titration (Figure 1; Supplementary Table 1). We measured higher mean IgG concentrations and NAb titers in subjects with a severe infection compared with those with a mild infection (Figure 3). All subjects with previous severe infection had NAbs for WT virus and the Alpha and Delta variants, but the proportion of subjects with NAbs was lower against Beta (80%) and Omicron BA.1 (33%) variants. The proportions of subjects with NAbs in the mild infection group were overall lower, reaching 79%, 50%, 21%, and 43% for WT virus, Alpha, Beta, and Delta variants, respectively. None of the subjects with a mild infection had NAbs against the Omicron BA.1 variant 8 months after infection (Table 2, Figure 3).

**Table 2.** Geometric Mean Titers, GMT (95% CI) of Neutralizing Antibody Titers to Wild-type Virus, Alpha (B.1.1.7), Beta (B.1.351), Delta (B.1.617.2), and Omicron (B.1.1.529/BA.1) Variants, and IgG Geometric Mean Concentrations, GMC (95% CI) Expressed as Binding Antibody Units/mL for WT SARS-CoV-2 Spike Proteins (RBD and SFL) 8 Months After Severe (n = 15) or Mild (n = 14) Infection and 1–3 Months After 1 Dose of COVID-19 Vaccine (Comirnaty) Administered 1 Year After the Infection; Geometric Mean Fold Changes Are Additionally Presented for Both NAb and IgG Data

	Severe Disease (n = 15)					Mild Disease (n = 14)				
	8 Mo After Infection		1–3 Mo After Vaccination			8 Mo After Infection		1–3 Mo After Vaccination		
	GMT (95% CI)		GMT (95% CI)		GMFC	GMT (95% CI)		GMT (95% CI)		GMFC
WT	44	(24–78)	1500	(660–3400)	34	6.9	(3.8–12)	180	(57–600)	27
Alpha	23	(15–37)	1100	(460–2400)	46	4.4	(2.6–7.4)	100	(29–360)	23
Beta	11	(5.7–21)	360	(150–870)	33	2.6	(1.8–3.8)	53	(16–180)	20
Delta	21	(13–35)	630	(280–1400)	30	3.7	(2.3–5.8)	75	(22–260)	21
Omicron	2.7	(2.1–3.5)	110	(46–240)	39	2.0	(2.0–2.0)	17	(7.4–37)	8.3
	GMC (95% CI)		GMC (95% CI)		GMFC	GMC (95% CI)		GMC (95% CI)		GMFC
WT RBD-IgG	15	(6.8–34)	154	(68–350)	10	0.51	(0.10–2.6)	41	(19–90)	83
WT SFL-IgG	13	(8.3–21)	340	(200–570)	26	0.90	(0.21–3.8)	84	(40–170)	94

Abbreviations: GMC, geometric mean concentration; GMFC, geometric mean fold change; GMT, geometric mean titer; IgG, immunoglobulin G; NAb, neutralizing antibody; RBD, receptor-binding domain; SARS-CoV-2, severe acute respiratory syndrome coronavirus 2; SFL, full-length spike protein; S-IgG, spike protein IgG; WT, wild-type.

One dose of Comirnaty among the subjects with previous infection induced very high mean IgG concentrations and NAb titers compared with the levels measured before vaccination (Table 2, Figure 3). We observed the highest mean NAb titers to the WT virus and reduced titers sequentially against Alpha, Delta, Beta, and Omicron BA.1 variants. Mean NAb titer fold changes ranged between 30 and 46 for severe and between 8 and 27 for mild disease depending on the virus strain (Table 2, Supplementary Figure 3). We found the most notable difference in fold change between the severe and mild infection groups for the Omicron BA.1 variant, suggesting that hybrid immunity against the Omicron BA.1 variant may be more enhanced after a severe compared with a mild infection. Subjects with a previous severe infection had NAbs to all virus strains analyzed (Figure 3). In the mild infection group, 1 dose of Comirnaty induced NAbs against WT virus in 100% of subjects, and positive or borderline positive titers were measured in a total of 86%, 79%, 86%, and 79% of the subjects for Alpha, Beta, Delta, and Omicron BA.1 variants, respectively. The higher level of IgG concentrations and NAb titers seen in subjects with a previous severe compared with mild infection remained after hybrid immunity (Table 2, Figure 3).

The NAb titers of 3 subjects within the mild infection group differed notably. None of them had detectable NAbs or IgG 8 months after infection. After vaccination, the IgG concentrations and NAb titers for the WT virus were detectable but markedly lower compared with the other subjects.

## DISCUSSION

Our study showed that a single COVID-19 vaccine dose induced strong SARS-CoV-2 spike-specific IgG and NAb responses in subjects with a previous infection, with 2-fold higher IgG levels compared with vaccinated subjects without previous infection. Additionally, we found a connection between disease severity and the development of hybrid immunity as we found that hybrid immunity was more enhanced after severe than mild infection. Further, we found that a second vaccine dose did not further enhance IgG antibody response induced by hybrid immunity and that a longer dosing interval (49–88 days) did not improve antibody response after the second dose. However, even with hybrid immunity, NAb titers remained reduced against heterologous variants, especially Omicron BA.1.

In line with our findings, other studies have also reported that hybrid immunity induced strong antibody responses [27–31], and comparable or superior responses compared with vaccinated individuals without previous infection [7, 8, 10, 16, 32]. We have previously shown that a single vaccine dose 3–6 months following WT, Alpha, or Beta infection induced S-IgG concentrations comparable to the levels following a third COVID-19 vaccine dose in previously uninfected individuals, whereas cross-reactivity of NAbs against different variants appeared to be even more enhanced [3]. Bates and coworkers found that even a mild breakthrough infection caused by the Delta variant substantially boosted humoral immunity induced by vaccination and improved variant cross-neutralization [33] and that antigen exposure from natural infection enhanced the magnitude and breadth of the antibody response similarly regardless of whether the exposure occurred before the vaccination (hybrid immunity) or after as a breakthrough infection [14]. This finding was also reported by Walls and coworkers, who showed that NAb responses were comparable in breakthrough cases, hybrid immunity, and in uninfected individuals vaccinated with 3 doses [34]. However, compared with previous studies, our finding on the impact of the severity of previous infection on the development of hybrid immunity is novel. Additionally, compared with previous studies, the strengths of our study include the study setting, with study subjects chosen initially as a random sample from a larger population, and that we assessed the antibody responses with the live-virus microneutralization test.

Our finding that a second vaccine dose after infection did not further increase antibody concentrations is in line with previous studies [8, 15, 35]. Moreover, a previous study conducted in Israel during the surge of the Delta variant in 2021 found no significant difference in vaccine effectiveness against reinfection after 1–2 doses of the vaccine [18]. However, Muecksch and coworkers showed that despite similar antibody concentrations after 2 or 3 doses of COVID-19 vaccine, the neutralizing potency and breadth of antibodies were increased after the third compared with the second vaccine dose in previously uninfected subjects [36], indicating that the third exposure to SARS-CoV-2 antigen expanded persisting clones of memory B cells expressing more potent and broader antibodies. Further, Wratil and coworkers reported that a second vaccination 9 months after infection further increased neutralization capacity against different variants including Omicron BA.1, suggesting that a longer dosing interval may be needed for optimal maturation of immunity and more efficient cross-variant neutralization [37]. Similarly, Miyamoto and coworkers found that a longer interval between vaccination and breakthrough infection was favorable for better antibody responses [38]. These findings suggest that maturation of the B-cell response takes place for several months following infection.

Despite our finding of higher immune responses in individuals with hybrid immunity than prior infection, breakthrough infections are possible also in individuals with hybrid immunity. However, hybrid immunity has been associated with a reduced risk of COVID-19-related symptoms [39], and the results of a recent meta-analysis of protection against Omicron re-infection strongly suggest that hybrid immunity appears to have more durable protection than prior infection alone [40].

In this study, the NAb titer pattern was similar to that seen in our previous studies [1, 3], with sequentially decreasing titers against the WT virus and the Alpha, Delta, Beta, and Omicron BA.1 variants. The lowest NAb titers against the Omicron BA.1 variant suggest reduced cross-protection against this previously circulated virus variant. None of the subjects with a mild WT infection had detectable NAbs against the Omicron BA.1 variant 8 months after the infection. We have also previously found that 36/37 subjects with a recent mild non-Omicron infection lacked NAbs against the Omicron BA.1 variant [3]. Recent studies have reported that Omicron infection induces stronger immune responses in previously vaccinated compared with naïve subjects [41] and that Omicron infection elicits NAbs that can cross-react with other sublineages of Omicron and other variants in individuals with hybrid immunity [42]. These findings emphasize the benefits of hybrid immunity and the need to vaccinate previously infected subjects to generate cross-reactive NAbs against different variants.

COVID-19 vaccination has been shown to induce lower antibody responses among individuals aged 65 and older [3, 9, 43, 44]. The relationship between age and antibody responses after infection or hybrid immunity is, however, more complex due to the greater overall disease severity among the elderly and higher antibody responses observed after a severe than a mild infection. Similarly, in this study, the median age of those with a severe infection was higher than that of those with a mild infection. Those with a severe infection had markedly higher NAb and IgG levels 8 months after WT infection, and the NAb titers against different variants remained at a significantly higher level up to 13 months following a severe compared with a mild WT infection [1]. In this study, we found that this difference persisted after a single dose of COVID-19 vaccination, suggesting enhanced duration of immunity and improved protection against emerging variants for those having experienced a severe infection.

Our study had some limitations. As this study was not designed to assess the kinetics of immune responses, we did not collect follow-up samples from the same individuals after vaccination, and the time points of sample collection were widespread and not always congruent. Also, because of the varying proportion of subjects with severe infection and age and gender distribution within groups, direct comparison of the effect of different dosing intervals or different vaccine preparations on hybrid immunity was not possible. Due to resource restrictions, we did not assess NAb titers against variants in hybrid immunity after 2 doses of vaccine and also did not assess neutralizing potencies of antibodies induced by hybrid immunity after 1 compared with 2 doses of the vaccine.

In summary, we found that a single dose of COVID-19 vaccine given a year after an infection strongly enhanced spike-specific IgG and NAb levels and neutralizing potency. NAb titers were higher in subjects with previous severe disease than mild disease and higher compared with vaccinated subjects without previous infection. The overall lowest NAb titers against Omicron BA.1 suggest reduced cross-protection against this variant in hybrid immunity. Our data support the importance of vaccinating both uninfected and previously infected individuals to elicit cross-variant neutralizing antibodies.

## Supplementary Material

ofac625_Supplementary_Data

## Supplementary Data

Supplementary materials are available at *Open Forum Infectious Diseases* online. Consisting of data provided by the authors to benefit the reader, the posted materials are not copyedited and are the sole responsibility of the authors, so questions or comments should be addressed to the corresponding author.
