# Supplementary material for: Strong Neutralizing Antibody Responses to SARS-CoV-2 Variants Following a Single Vaccine Dose in Subjects With Previous SARS-CoV-2 Infection
Source: Open Forum Infect Dis. 2022 Nov 19;9(12):ofac625. doi: 10.1093/ofid/ofac625 (PMC9745780; doi:10.1093/ofid/ofac625)
Supplement: ofac625_Supplementary_Data [file ofac625_supplementary_data.docx]

**Strong neutralizing antibody responses to SARS-CoV-2 variants following a single vaccine dose in subjects with previous SARS-CoV-2 infection**

Nina Ekström, Anu Haveri, Anna Solastie, Camilla Virta, Pamela Österlund, Hanna Nohynek, Tuomo Nieminen, Lauri Ivaska, Paula A. Tähtinen, Johanna Lempainen, Pinja Jalkanen, Ilkka Julkunen, Arto A. Palmu, and Merit Melin

**OFID_Supplementary material**

**Supplementary-Figure 1.** SARS-CoV-2 spike specific IgG antibody concentrations for the receptor binding domain, RBD (A) and full-length spike protein, SFL (B) expressed as binding antibody units BAU/ml (GMC, 95% CI) for samples taken prior to the first vaccine dose and 7 to 30, 31 to 60 and 61 to 90 days after the last vaccine dose (Comirnaty or Vaxzevria). Samples collected at different time points were not collected from the same subjects.

**
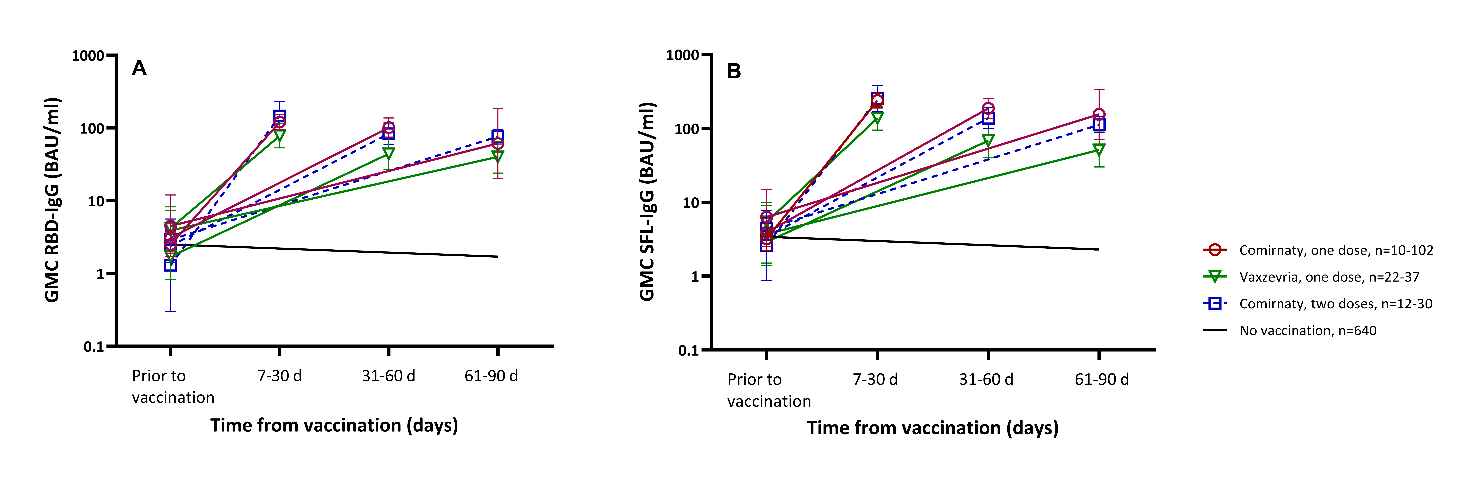
**

**Supplementary Figure 2.** SARS-CoV-2 spike specific IgG antibody concentrations for the receptor binding domain, RBD (A) and SFL- full-length spike protein, SFL (B) expressed as binding antibody units BAU/ml (GMC, 95% CI) in subjects with or without a previous SARS-CoV-2 infection and after one or two doses of COVID-19 vaccine (Comirnaty or Vaxzevria). Samples from subjects with previous severe infection are shown by open labels. Two vaccine doses were administered either with a short dosing interval^#^ (median of 21 days apart (range 19-28) or a long dosing interval* (median of 84 days apart, range 49-88 days). Statistically significant differences between the most relevant groups are indicated with asterisks (Wilcoxon rank-sum test) ns = not significant, * = *P*<.05, ** = *P*<.01, *** = *P*<.001, **** =*P*<.0001

**
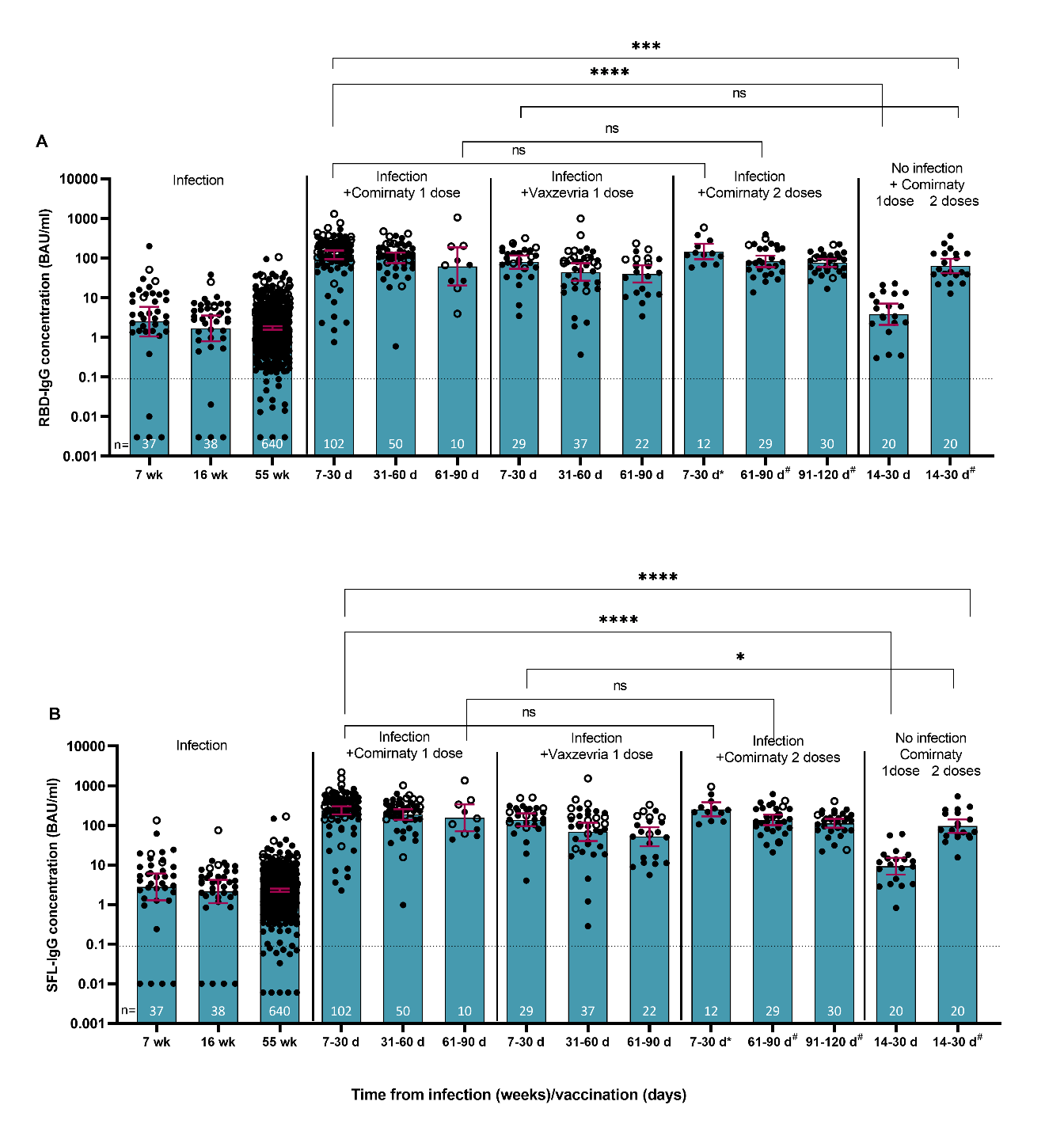
**

**Supplementary Figure 3**. Mean fold changes in neutralizing antibody titers to wild-type (wt) virus, Alpha (B.1.1.7), Beta (B.1.351), Delta (B.1.617.2) and Omicron (B.1.1.529/BA.1) variants in severe (n=15) or mild (n=14) infection groups between samples taken before vaccination (eight months after infection) and 1 to 3 months after one dose of COVID-19 vaccine (Comirnaty) administered one year after infection. The height of the bars represents medians and whiskers the interquartile range within infection group. Statistically significant differences between groups of mild and severe infection are indicated with asterisks (Wilcoxon rank-sum test) ns = not significant, * = *P*<.05, ** = *P*<.01, *** = *P*<.001.

**
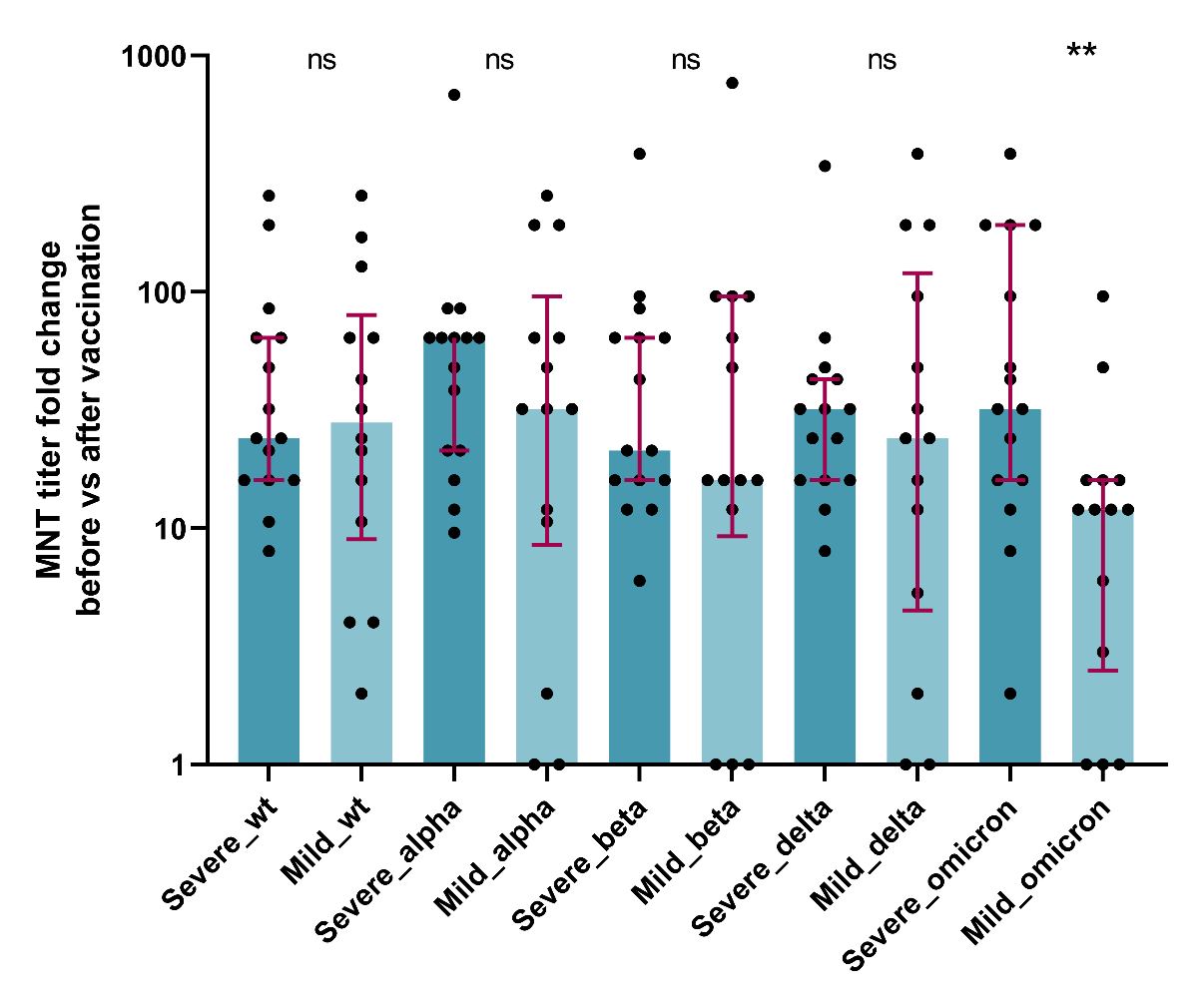
**

**Supplementary Table 1.** Characteristics of the participants in the study subgroups and timing of vaccination and serum sampling

| **Infection and vaccination status,** **days from vaccination or infection** | **n** | **Days from infection to vaccination,** **median (range)** | | **Age,** **median (range)** | | **Severe disease**  **n (%)** | | | **Female (%)** |
| --- | --- | --- | --- | --- | --- | --- | --- | --- | --- |
| ***Infected, Comirnaty, one dose*** |  |  |  |  |  | |  |  |  |
| 7-30 | 102 | 369 | (342-424) | 62 | (26-79) | | 23 | (23) | 58 |
| 31-60 | 50 | 353 | (312-419) | 73 | (30-96) | | 11 | (22) | 64 |
| 61-90 | 10 | 310 | (299-334) | 77 | (29-90) | | 6 | (60) | 60 |
| ***Subset selected for NAb titration*** |  |  |  |  |  | |  |  |  |
| 22-85 (severe infection) | 15 | 338 | (307-377) | 77 | (69-97) | | 15 | (100) | 33 |
| 22-83 (mild infection) | 14 | 355 | (299-415) | 52 | (28-76) | | 0 | (0) | 64 |
|  |  |  |  |  |  | |  |  |  |
| ***Infected, Vaxzevria, one dose*** |  |  |  |  |  | |  |  |  |
| 7-30 | 29 | 372 | (349-399) | 67 | (62-73) | | 13 | (45) | 38 |
| 31-60 | 37 | 340 | (313-360) | 57 | (28-67) | | 13 | (35) | 62 |
| 61-90 | 22 | 322 | (290-351) | 56 | (38-67) | | 6 | (27) | 73 |
|  |  |  |  |  |  | |  |  |  |
| ***Infected, Comirnaty, two doses*** |  |  |  |  |  | |  |  |  |
| 7-30^#^ | 12 | 292 | (267-313) | 52 | (27-66) | | 1 | (8) | 92 |
| 61-90^*^ | 29 | 280 | (261-293) | 52 | (26-62) | | 2 | (7) | 83 |
| 91-120^*^ | 30 | 270 | (250-314) | 56 | (25-91) | | 4 | (13) | 90 |
|  |  |  |  |  |  | |  |  |  |
| ***Infected, not vaccinated*** |  |  |  |  |  | |  |  |  |
| 14-60 | 37 | - | - | 47 | (23-80) | | 3 | (8) | 59 |
| 90-148 | 38 | - | - | 47 | (23-80) | | 3 | (8) | 63 |
| 362-448 | 640 | - | - | 48 | (18-81) | | 78 | (12) | 59 |
|  |  |  |  |  |  | |  |  |  |
| ***Uninfected, Comirnaty*** |  |  |  |  |  | |  |  |  |
| 14-30 (after one dose) | 20 | - | - | 42 | (25-61) | | - | - | 90 |
| 14-30 (after two doses) | 20 | - | - | 42 | (25-61) | | - | - | 90 |

#1^st^ and 2^nd^ vaccine at a median of 84 days apart (range 49-88)
*1^st^ and 2^nd^ vaccine at a median of 21 days apart (range 19-28)

**Supplementary Table 2.** Details of the SARS-CoV-2 virus strains used in live-virus microneutralization test

| **WHO label** | **Pango lineage** | **Isolate** | **GISAID accession ID** | **GenBank accession ID** |
| --- | --- | --- | --- | --- |
| **Wild-type** | B | hCoV-19/Finland/1/2020 | EPI_ISL_407079 | MZ934691 |
| **Alpha** | B.1.1.7 | hCoV-19/Finland/THL-202102301/2021 | EPI_ISL_2590786 | MZ944886 |
| **Beta** | B.1.351 | hCoV-19/Finland/THL-202101018/2021 | EPI_ISL_3471851 | MZ944846 |
| **Delta** | B.1.617.2 | hCoV-19/Finland/THL-202117309/2021 | EPI_ISL_2557176 | MZ945494 |
| **Omicron/BA.1** | B.1.1.529 | hCoV-19/Finland/THL-202126660/2021 | EPI_ISL_8768822 | OM393712 |
